# RNA-Seq identifies condition-specific biological signatures of ischemia-reperfusion injury in the human kidney

**DOI:** 10.1186/s12882-020-02025-y

**Published:** 2020-09-25

**Authors:** Meeyoung Park, Chae Hwa Kwon, Hong Koo Ha, Miyeun Han, Sang Heon Song

**Affiliations:** 1 Biomedical Research Institute, Pusan National University Hospital, Busan, South Korea; 2 Department of Urology, Pusan National University Hospital, Busan, South Korea; 3 Department of Internal Medicine and Biomedical Research Institute, Pusan National University Hospital, Busan, South Korea

**Keywords:** Acute kidney injury, Ischemia-reperfusion injury, Clustering analysis, Pathway analysis, RNA-sequencing

## Abstract

**Background:**

Acute kidney injury (AKI) is defined as a sudden event of kidney failure or kidney damage within a short period. Ischemia-reperfusion injury (IRI) is a critical factor associated with severe AKI and end-stage kidney disease (ESKD). However, the biological mechanisms underlying ischemia and reperfusion are incompletely understood, owing to the complexity of these pathophysiological processes. We aimed to investigate the key biological pathways individually affected by ischemia and reperfusion at the transcriptome level.

**Results:**

We analyzed the steady-state gene expression pattern of human kidney tissues from normal (pre-ischemia), ischemia, and reperfusion conditions using RNA-sequencing. Conventional differential expression and self-organizing map (SOM) clustering analyses followed by pathway analysis were performed. Differential expression analysis revealed the metabolic pathways dysregulated in ischemia. Cellular assembly, development and migration, and immune response-related pathways were dysregulated in reperfusion. SOM clustering analysis highlighted the ischemia-mediated significant dysregulation in metabolism, apoptosis, and fibrosis-related pathways, while cell growth, migration, and immune response-related pathways were highly dysregulated by reperfusion after ischemia. The expression of pro-apoptotic genes and death receptors was downregulated during ischemia, indicating the existence of a protective mechanism against ischemic injury. Reperfusion induced alterations in the expression of the genes associated with immune response such as inflammasome and antigen representing genes. Further, the genes related to cell growth and migration, such as *AKT, KRAS*, and those related to Rho signaling, were downregulated, suggestive of injury responses during reperfusion. Semaphorin 4D and plexin B1 levels were also downregulated.

**Conclusions:**

We show that specific biological pathways were distinctively involved in ischemia and reperfusion during IRI, indicating that condition-specific therapeutic strategies may be imperative to prevent severe kidney damage after IRI in the clinical setting.

## Background

In the kidney, ischemia-reperfusion injury (IRI) is characterized with the temporary deficiency of oxygen due to restricted blood flow, followed by the sudden restoration of oxygen supply. The result is acute kidney injury (AKI), which may vary from a subtle kidney dysfunction to the need for renal replacement therapy [1]. Physiologically, approximately 66% AKI are induced by IRI or acute tubular necrosis [2, 3]. According to a recent meta-analysis of 154 studies based on the strict definition by Kidney Disease: Improving Global Outcomes (KDIGO), 23% AKI incidence occur during hospitalization and mortality is reported in approximately 50–80% patients with severe AKI [4]. Hence, interventions such as continuous renal replacement therapy have been increasingly adopted as a treatment strategy in patients with severe AKI, which has evolved into a socioeconomic burden [5].

AKI is closely interconnected and integrated with chronic kidney disease (CKD). AKI is a risk factor of incidence of CKD, which itself is a risk factor of AKI episodes [6, 7]. Moreover, AKI-induced CKD is most likely to progress to stage 4 CKD and decrease survival time [6]. The continuation of the inflammatory response of the kidney tissue following AKI results in incomplete recovery and accelerates the process of injury, thereby provoking CKD. Therefore, the understanding of the mechanism underlying the development of AKI is critical to prevent its progression into ESKD.

The various pathophysiological characteristics of AKI pose difficulties to evaluate the underlying mechanism, thereby contributing to poor patient prognosis [8]. For instance, serum creatinine level may not completely reflect the loss of kidney function during early stages [9]. Further, considering the ethical and regulatory obstacles related to human clinical studies, many studies on AKI have been conducted as observational or treatment research using murine animals [10–13]. These animal models of AKI mostly include young male mice with normal kidney functions that are evidently different from the actual clinical condition in human patients [14]. In addition, it is rather difficult to obtain pre-hypoxic kidney tissues from healthy humans for comparative and analytical purposes. Together these reasons have hindered research on human AKI and obstructed the development of treatment strategies for the prevention of AKI caused by IRI.

In the present study, we investigated the key genes and biological pathways affected separately after ischemia and reperfusion during IRI in human kidney tissues. We performed RNA sequencing (RNA-seq) to examine changes in the gene expression pattern via conventional differential gene expression and self-organizing map (SOM) clustering analyses [15]. The important contributions of our study are as follows: First, no study has been conducted on IRI using human kidney tissues. Therefore, we believe that our findings could improve our understanding of the mechanisms of IRI in humans. Second, this is the first study to perform transcriptome analysis using next-generation sequencing such as RNA-seq separately in ischemia and reperfusion within a short period, although a recent study reported RNA-seq result before and after kidney transplantation [16]. Our study allows us to understand the biological mechanism of ischemia and reperfusion through the analysis of the whole transcriptome data obtained for the human kidney tissue. Third, the time-series IRI tissue analysis facilitates the identification of the expression trajectory of the key genes affected by ischemia and reperfusion. Lastly, machine learning algorithms help us to broaden our knowledge by highlighting the expression patterns of specific genes of interest from large-scale gene expression data during IRI.

We reveal the specific genes and pathways that are involved separately in ischemia and reperfusion during IRI in the human kidney. We suggest that a condition-specific therapeutic approach may be imperative for the effective prevention of severe kidney damage after IRI in the clinical setting. Further investigations are warranted to understand the functions of the newly discovered biological signatures related to IRI.

## Methods

### Patients

**Fig. 1:**
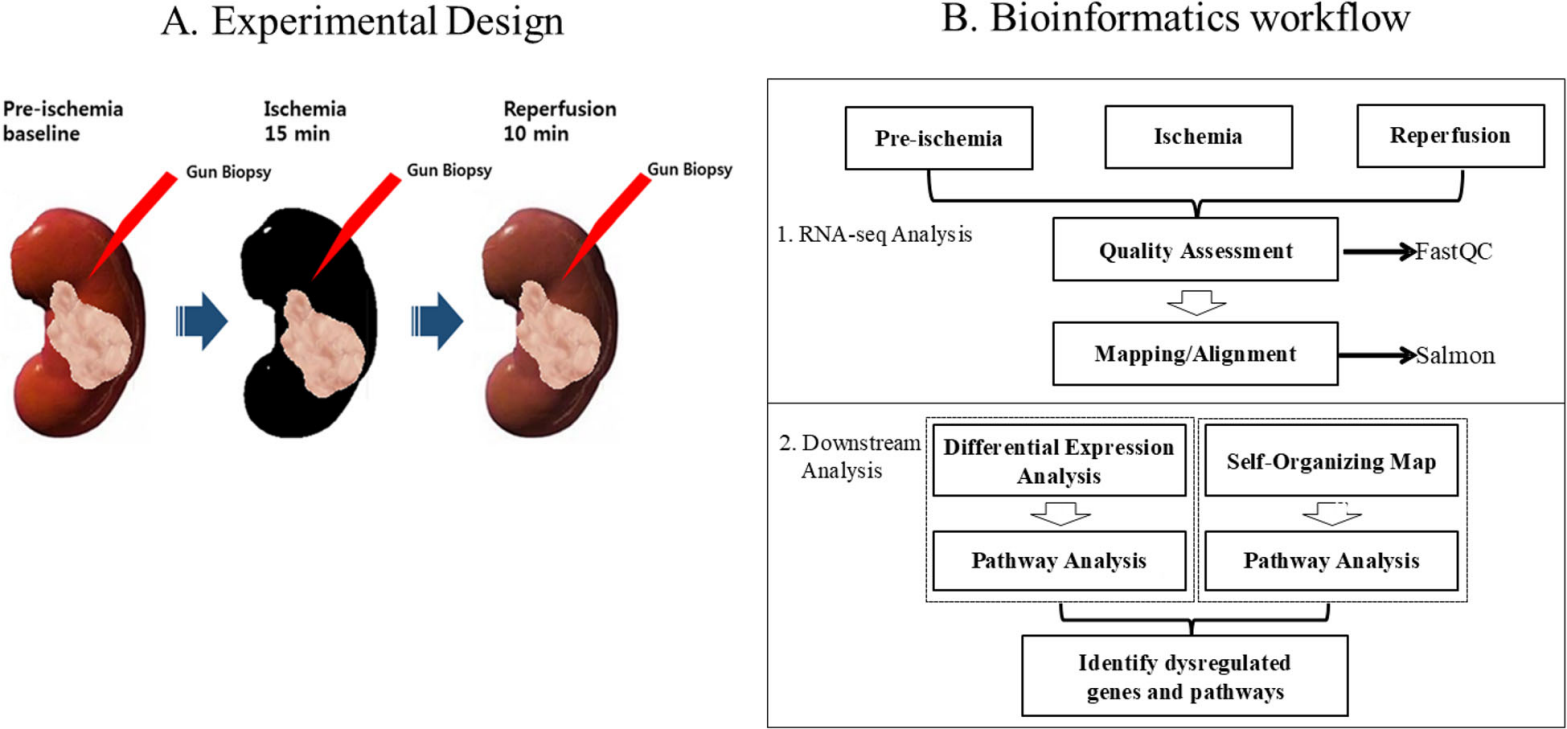
Overall experimental design and workflow. **a** Kidney cortex tissues were obtained from five male patients with kidney cell carcinoma or transitional cell carcinoma scheduled for total nephrectomy. The base-line expression was analyzed under normal condition or pre-ischemia. Gun biopsy was performed for ischemia after 15 min of ischemia and 10 min later, the biopsy was repeated for reperfusion. **b** Bioinformatic workflow: FastQC for quality assessment and Salmon were executed for RNA-seq quantification from pre-ischemia, ischemia, and reperfusion conditions. Differential expression analysis and self-organizing map (SOM) followed by pathway analysis were performed in parallel to identify key genes and pathways significantly associated with ischemia-reperfusion

Five male patients scheduled for total nephrectomy owing to renal cell carcinoma or transitional cell carcinoma were enrolled in the study. Their average age was 64.8 years and their kidney functions before surgery were near normal state (mean creatinine: 0.89 mg/dL, mean estimated glomerular filtration rate [eGFR]: 88.1 mL·min·^− 1^1.73 m^− 2^, mean hemoglobin: 14.2 g/dL) (Supplementary Table S1). The kidney cortical tissue was obtained by gun biopsy at three time points as follows: pre-ischemia (considered as a normal condition), ischemia (after 15 min of hypoxia), and after 10 min of reperfusion (Fig. 1a). The tissue samples were immediately transferred to individual cryotubes prefilled with 0.5 mL RNAlater® (QIAGEN Inc., Hilden, Germany) and stored at room temperature for 24 h. RNAlater® was removed following incubation, and tissues was stored at − 80 °C according to the manufacturer’s instructions until analysis. Our study protocol was approved by the Pusan National University Hospital Ethics Committee (IRB number H-17020002-051). All participants provided written informed consent as requested by our Ethics Committee, and all procedures were implemented in accordance with the Helsinki Declaration.

### RNA-seq analysis

To identify the changes induced by ischemia and reperfusion at the transcriptomic level, we analyzed the steady-state gene expression pattern during pre-ischemia (normal), ischemia, and reperfusion using RNA-seq (Fig. 1b). Total RNA was isolated from the kidney cortex of five male patients at each condition using mirVana™ miRNA Isolation Kit (ThermoFisher, Inc., Seoul, Korea). The RNA quality was assessed using 2100 Expert Bioanalyzer with RNA 6000 Nano Kit (Agilent, Inc., Santa Clara, CA, USA). Samples with RNA integrity number > 7 were prepared using Illumina TruSeq Standard mRNA Prep kit (Catalog #RS-122-2103; Illumina, San Diego, CA, USA). After quantitative polymerase chain reaction (qPCR) using SYBR Green PCR Master Mix (Applied Biosystems), the libraries were combined such that the indexed sample was present at equimolar concentrations in the pool. Cluster generation was carried out in the flow cell on the cBot automated cluster generation system (Illumina). The flow cell was loaded on HiSeq 2500 sequencing system (Illumina), and sequencing was performed with 2× 100 bp read length. RNA-seq was carried out by DNA Link, Inc., Seoul, Korea (http://www.dnalink.com/).

FastQC (http://www.bioinformatics.babraham.ac.uk/projects/fastqc/) was conducted to assess the quality of RNA-seq data. The RNA-seq data were quantified using an alignment-free tool, Salmon, developed by Patro *et. al* in 2017 that estimates the relative abundance of all transcripts [17]. GRCh38.p13 was used as the reference transcriptome to quantify read counts. The general information of RNA-seq is shown in Supplementary Table S2, and RNA-seq data are available in Gene Expression Omnibus (GEO) database under the accession number GSE142077.

### Differential expression analysis

We employed *tximport* [18] with Bioconductor differential gene expression package using R (version 3.5.1) to assemble count values from each sample. The assembled count values were used as the input of DESeq2 Bioconductor package [19]. The significantly differentially expressed genes (DEGs) between groups were defined at cut-off criteria of |log_2_ fold-change| ≥ 1 and *p*-value < 0.05. Significantly enriched pathways were examined for the identified DEG sets.

### Self-organizing map analysis

To identify the trajectory patterns of gene expression across all three time points, pre-ischemia, ischemia, and reperfusion, SOM clustering [15] analysis was performed. A 7 × 7 grid panel was selected for the SOM output structure to intuitively interpret the results. Transcripts showing similar expression patterns across three conditions were gathered in a module. Selecting rules were applied to 49 modules to obtain modules of interest. Selected modules with similar expression patterns across three groups were combined as a ‘cluster.’ SOM algorithm implemented in MATLAB 2018b software (http://www.mathworks.com) was used. Genes in each cluster were further analyzed to identify significantly enriched canonical pathways.

### Pathway analysis

The Ingenuity Pathway Analysis (IPA) software (www.qiagen.com/ingenuity, Spring 2019, QIAGEN, CA, USA) was used to identify enriched biological pathways. A *p*-value or Benjamini-Hochberg adjusted *p*-value was calculated using Fisher’s exact test, and a cut-off value of less than 0.05 was used to identify significantly enriched canonical pathways based on the Ingenuity Knowledge Base.

## Results

### Study workflow

We aimed to identify the key genes and pathways through the evaluation of gene expression changes under pre-ischemia (normal), ischemia, and reperfusion conditions in human kidney samples using RNA-seq (Fig. 1b). RNA-seq was performed for downstream bioinformatic analyses, namely, differential gene expression and SOM clustering analyses. Differential expression analysis was performed to identify the significant DEGs between two conditions. SOM clustering was carried out to group the whole gene expression data and identify specific patterns of interest across all three groups without prior knowledge. Finally, the selected genes of interest from both analyses were used as inputs for pathway analysis and to identify the most significantly affected biological pathways separately under ischemia and reperfusion during IRI.

### Differential expression analysis

**Fig. 2:**
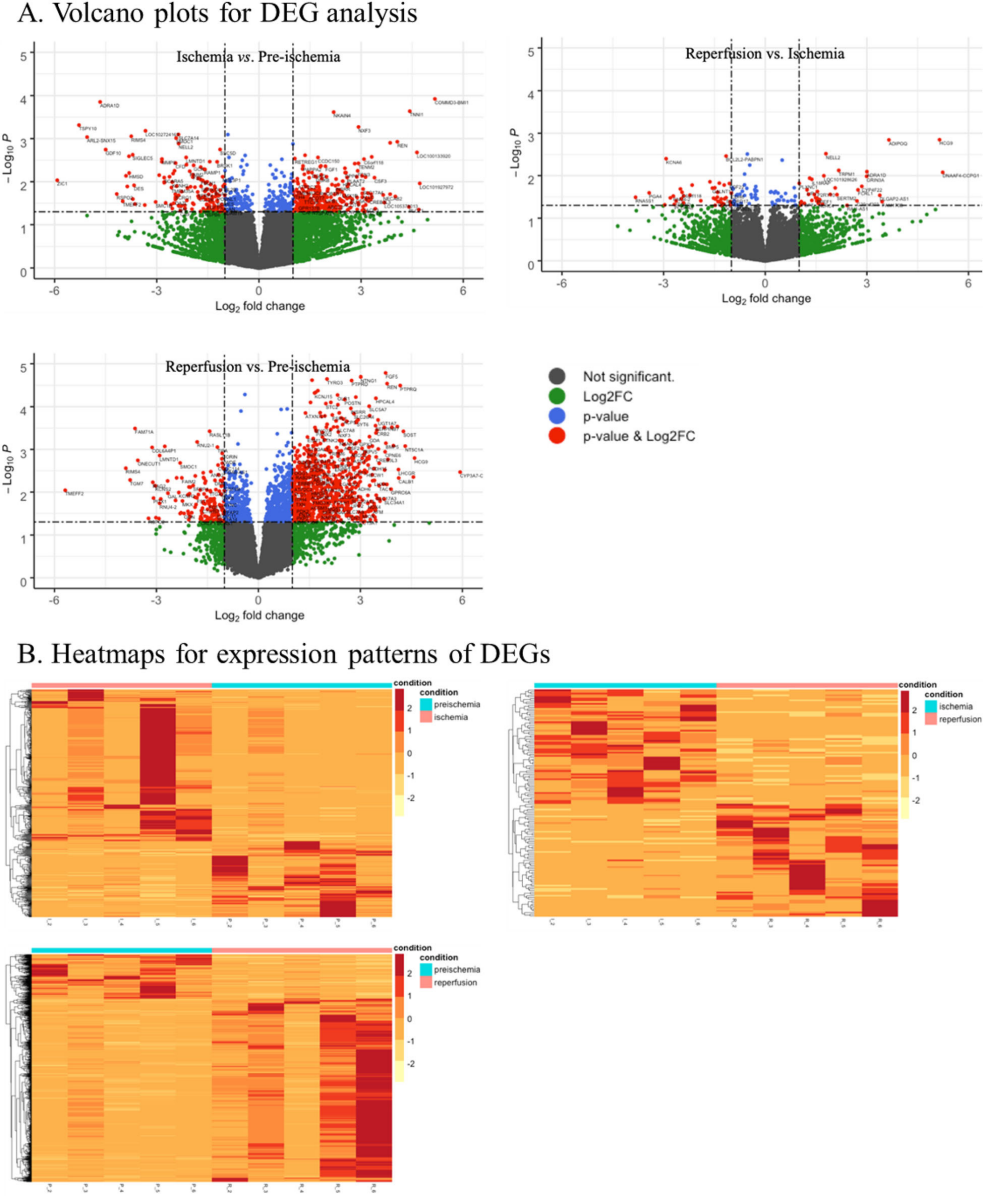
Differential expression analysis. We evaluated the differentially expressed genes (DEGs) for three groups: ischemia versus pre-ischemia, reperfusion versus ischemia, and reperfusion versus pre-ischemia. We used |log_2_ fold-change| ≥ 1 and *p*-value < 0.05 as criteria to identify significant DEGs between the groups. The volcano plots show all the DEGs for each comparison group (**a**). The heatmaps display the expression patterns of significant DEGs (**b**)

Differential expression analysis is a conventional method for the identification of quantitative changes in the expression levels of genes between two groups. We evaluated the DEGs by performing three comparisons as follows: (1) ischemia versus pre-ischemia to identify the genes affected by ischemia; (2) ischemia versus reperfusion to detect the genes affected by reperfusion after ischemia; (3) reperfusion versus pre-ischemia to analyze the genes affected by the complete IRI process. We used |log_2_ fold-change| ≥ 1 and *p*-value < 0.05 as criteria to identify significant DEGs between the groups (Fig. 2).

As a result, 603 DEGs (upregulated: 402 genes; downregulated: 201 genes) were significantly dysregulated between ischemia and pre-ischemia samples and 135 DEGs (upregulated: 67 genes; downregulated: 68 genes) were found to be significantly dysregulated between reperfusion and ischemia samples. However, 1389 DEGs (upregulated: 1119 genes; downregulated: 270 genes) were dysregulated between reperfusion and pre-ischemia conditions. The top 20 DEGs in each comparison group are shown in Supplementary Tables S3-S5.

**Fig. 3:**
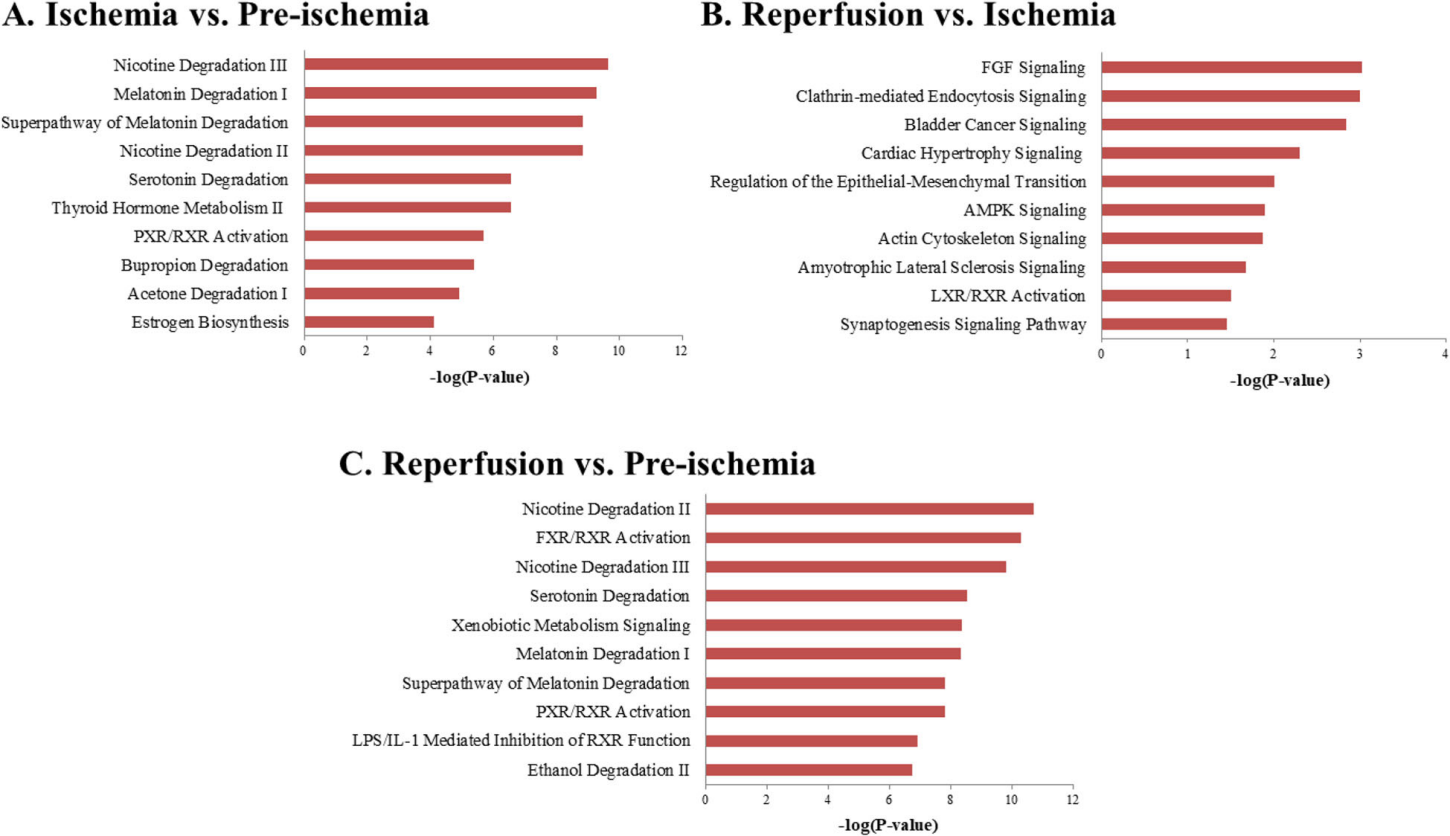
Enriched pathways associated with DEGs. We analyzed the enriched pathways of DEGs for three groups using Ingenuity Pathway Analysis: ischemia versus pre-ischemia (**a**), reperfusion versus ischemia (**b**), and reperfusion versus pre-ischemia (**c**). The top 10 most significantly enriched pathways are presented

To determine the pathways associated with ischemia and reperfusion, the IPA was performed to investigate the biological pathways associated with the DEG sets. The top pathways related to the DEGs between ischemia versus pre-ischemia conditions were mainly metabolic pathways and included genes encoding cytochrome P450 enzymes (*CYP1A2*, *CYP2C8*, *CYP2C9*, *CYP2J2*, *CYP3A4*, *CYP3A7*). Uridine diphosphate glucuronosyltransferase (*UGT1A1*, *UGT1A6*, *UGT1A7*, *UCT2A3*, *UGT2B11*, *UGT3A1*) is involved in the metabolism of various molecules, including steroids, hormones, and drugs (Fig. 3a and Supplementary Table 6) [20, 21]. Ischemia induced changes in metabolites such as nicotine, melatonin, serotonin, and thyroid hormone. Melatonin and serotonin are known to exert antioxidant proprieties under oxidative stress [22], and may protect the function of the kidney during early ischemic injury. In addition, drug metabolism pathways, including bupropion, acetone degradation and estrogen biosynthesis, and pregnane X receptor/retinoic X receptor (PXR/RXR) pathway were related to ischemic process. PXR is a nuclear receptor activated by endogenous compounds and clinical drugs. Activated PXR in conjunction with RXR plays a central role in drug metabolism by inducing the expression of the cytochrome P450 family. This receptor was recently reported to be involved in inflammation, proliferation, and apoptosis [23].

The DEGs between reperfusion and ischemia conditions were found to be enriched in cellular assembly, development and migration, energy production, and inflammasome-related signaling pathways (Fig. 3b and Supplementary Table 7). Fibroblast growth factor (FGF) performs diverse functions through the activation of several pathways by binding to FGF receptor (FGFR). FGF signaling pathway is involved in cellular assembly and development and migration, including hypertrophy, regulation of epithelial-mesenchymal transition, and actin cytoskeleton signaling, which are affected in response to renal ischemia/reperfusion [24, 25]. AMP-activated protein kinase (AMPK) involved in metabolic processes generating ATP and liver X receptor (LXR)/RXR signaling associated with lipid metabolism were also detected. In addition, inflammasome pathway was enriched, indicating that reperfusion may induce an immune response.

The comparison between reperfusion and pre-ischemia (reperfusion versus pre-ischemia) conditions showed that reperfusion induced changes in various pathways related to lipid and drug metabolism such as nicotine, serotonin, and melatonin degradation, FXR/RXR, and PXR/RXR signaling pathway, consistent with the results between ischemia and pre-ischemia conditions (Fig. 3c and Supplementary Table 8). Different pathways are involved in tryptophan, valine, ethanol, and histamine degradation as well as fatty acid oxidation. These results suggest that reperfusion affected not only lipid and drug metabolism but also energy metabolism. In summary, the pathway analysis for DEG sets showed that metabolic pathways were affected under both ischemia and reperfusion conditions. In particular, reperfusion affected hypertrophy, cellular assembly and development, and inflammatory response-related signaling pathways.

### SOM clustering analysis

**Fig. 4:**
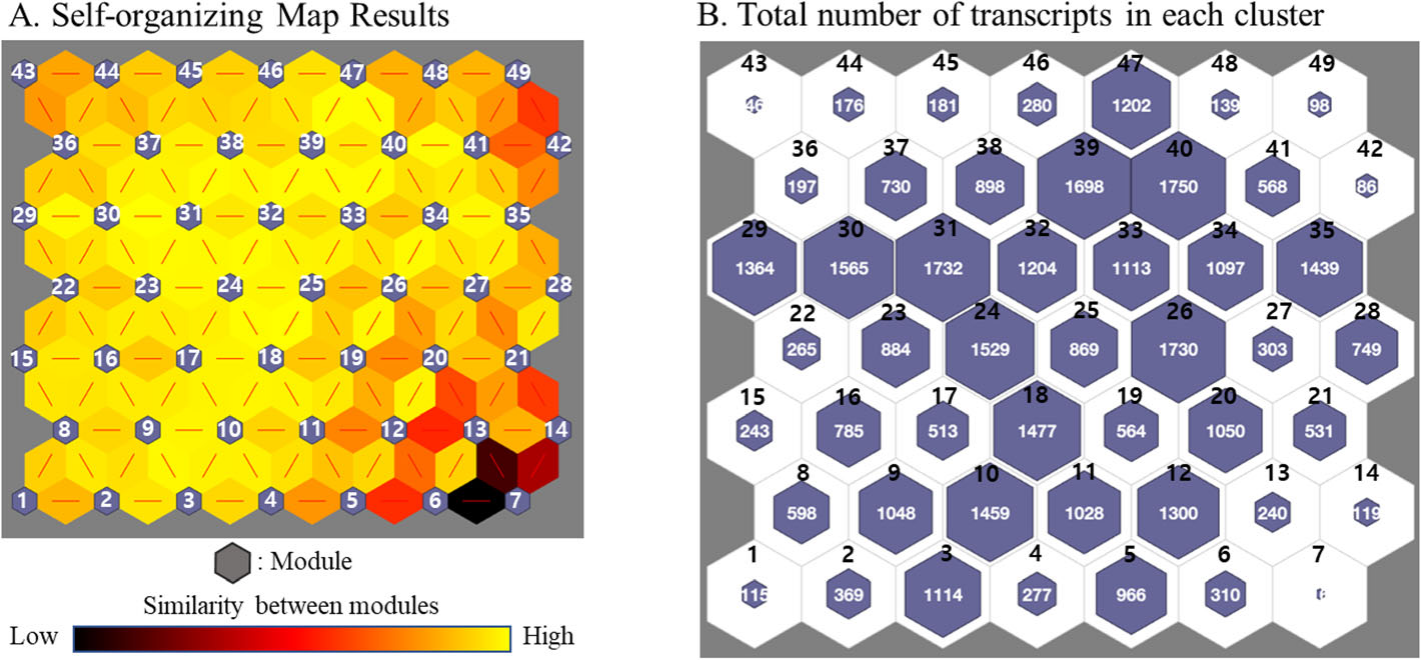
SOM analysis results. **a** The 7 × 7 grid structure was chosen for SOM output to enhance interpretability. The results of SOM were visualized as a colored grid panel with blue hexagons and similarity color between the hexagons. We defined the hexagon as a ‘module,’ and all genes included in each module showed a similar expression pattern. The similarity between adjacent modules was represented with a yellow-black color scheme; a color close to yellow indicated the similar expression pattern between adjacent modules, while a color close to black indicated the distinct patterns between adjacent modules. **b** Each module contained transcripts with similar expression patterns across pre-ischemia, ischemia, and reperfusion conditions. The total number of transcripts in each module is shown

Among the clustering algorithms such as hierarchical clustering or *k*-means clustering, SOM has been extensively used for the analysis of large-scale gene expression data [26–31]. We performed SOM clustering analysis to examine gene expression patterns across all three conditions. Transcripts per million (TPM) is a commonly used normalization method as previously described in [32]. The log2-transformed TPM values of transcripts for each group were used as SOM input data. Given the small sample size, we calculated median values of log2-transformed TPM for each group. However, as technical and biological biases often generate inexplicable results, we removed zero expression values and filtered the very low signal values (< log_2_3) for further analyses. As a result, 38,014 transcripts were used for SOM input. The 7 × 7 grid structure was chosen for SOM output to enhance biological interpretability. The results were visualized as a colored grid panel with blue hexagons and a yellow-black similarity color scheme between the hexagons (Fig. 4a). We defined hexagon as a ‘module’ such that the genes included in each module showed a similar expression pattern across all three conditions. The similarity between adjacent modules was represented with different colors; a color close to yellow was indicative of the similar expression pattern between adjacent modules. On the contrary, a color close to black was suggestive of the distinct patterns of the adjacent modules. The total number of transcripts in each module after clustering is shown in Fig. 4b.

**Fig. 5:**
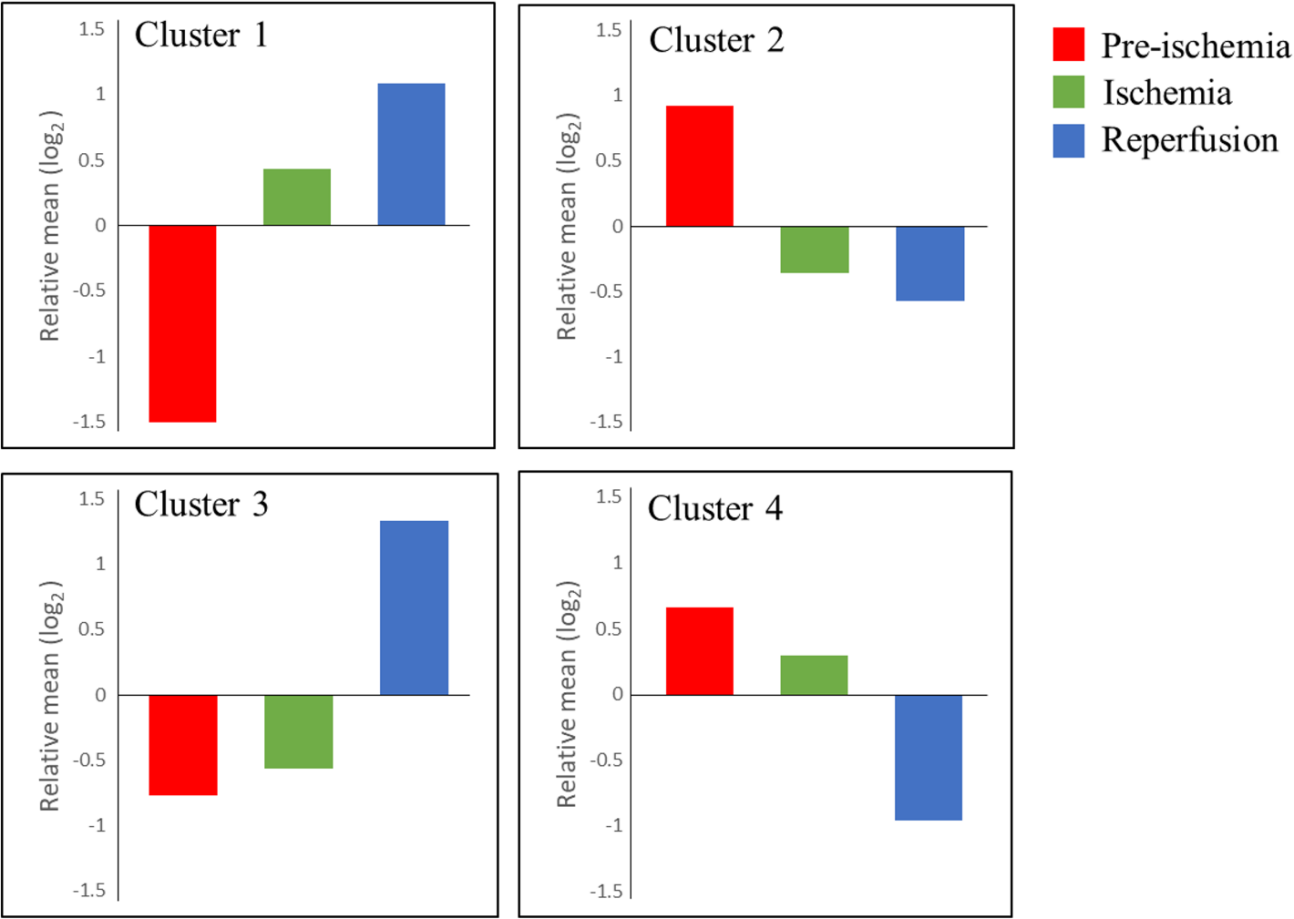
Expression patterns of selected clusters. Genes in Cluster 1 and 2 were upregulated or downregulated in ischemia and reperfusion as compared with those in pre-ischemia condition. Genes in Cluster 3 and 4 were upregulated or downregulated after reperfusion as compared with those in pre-ischemia and ischemia. The vertical axis was represented as the relative mean of the cluster for the conditions

As we aimed to determine the change in the expression pattern of genes under each condition, we focused on the specific patterns of gene expression under ischemia (genes upregulated or downregulated in ischemia versus pre-ischemia) and reperfusion (genes upregulated or downregulated in reperfusion versus ischemia). Modules of interest among 49 modules were selected based on the criteria rules (Table 1). As a result, module 42, 44, 45, 46, 48, and 49 including the genes upregulated in ischemia were defined as ‘Cluster 1.’ Module 1, 8, 11, 17, and 19 carrying the genes downregulated during ischemia were defined as ‘Cluster 2.’ Similarly, the genes in modules 36 and 43 upregulated by reperfusion as compared to ischemia and pre-ischemia were defined as ‘Cluster 3.’ Lastly, the genes in module 15, 16, and 23 that were downregulated in reperfusion were defined as ‘Cluster 4’ (Fig. 5). In total, 3035 and 1917 genes were affected in ischemia and reperfusion, respectively (Table 2).

**Table 1:** Condition-specific criteria for selecting modules of interest among 49 modules

Condition	Expression pattern	Criteria
Ischemia effect	Upregulated	${\left\{\left[{\mathit{\log}}_2\left(\overline{I}/\overline{P}\right)\right]\right\}}_{module_i}\ge 1\ AND\ {\left\{\left[{\mathit{\log}}_2\left(\overline{R}/\overline{P}\right)\right]\right\}}_{module_i}\ge 1$
	Downregulated	${\left\{\left[{\mathit{\log}}_2\left(\overline{I}/\overline{P}\right)\right]\right\}}_{module_i}\le 1\ AND\ {\left\{\left[{\mathit{\log}}_2\left(\overline{R}/\overline{P}\right)\right]\right\}}_{module_i}\le 1$
Reperfusion effect	Upregulated	${\left\{\left[{\mathit{\log}}_2\left(\overline{R}/\overline{I}\right)\right]\right\}}_{module_i}\ge 1\ AND\ {\left\{\left[{\mathit{\log}}_2\left(\overline{R}/\overline{P}\right)\right]\right\}}_{module_i}\ge 1$
	Downregulated	${\left\{\left[{\mathit{\log}}_2\left(\overline{R}/\overline{I}\right)\right]\right\}}_{module_i}\le 1\ AND\ {\left\{\left[{\mathit{\log}}_2\left(\overline{R}/\overline{P}\right)\right]\right\}}_{module_i}\le 1$

$\overline{P,}\overline{I},\overline{R}:$ the mean value of TPM for the genes in pre-ischemia, ischemia, and reperfusion groups in a module, respectively, *i* = 1..49

**Table 2:** Condition-specific cluster information

Clusters		Module number	Number of genes	Total number of genes
Ischemia effect	Cluster 1 (Upregulated)	42, 44, 45, 46, 48, 49	633	3035
	Cluster 2 (Downregulated)	1, 8, 11, 17, 19	2402	
Reperfusion effect	Cluster 3 (Upregulated)	36, 43	227	1917
	Cluster 4 (Downregulated)	15, 16, 23	1690	

We performed pathway analysis using IPA for the selected clusters to identify significantly enriched pathways. The genes dysregulated by ischemia (Cluster 1 and 2) were mainly enriched in apoptosis-related pathways, including aryl hydrocarbon receptor and death receptor signaling pathway (Table 3). During ischemia, FAS cell surface death receptor (*FAS*) and tumor necrosis factor (*TNF*) related to complex signaling pathways for cell death were down-regulated, suggestive of the presence of a protective mechanism against cell death. In addition, intracellular and secondary messenger signaling pathways such as protein ubiquitination, adipogenesis, and apelin adipocyte signaling pathway were identified. Lipid accumulation and deposition is known to induce lipotoxicity, thereby leading to ischemia-mediated kidney injury [27]. Further, metabolism-related pathways such as PXR/RXR signaling pathway and xenobiotic metabolism signaling were enriched. PXR/RXR signaling pathway was also detected in DEG analysis.

**Table 3:** Top 10 most significantly enriched pathways in Cluster 1 and 2 using IPA^a^

Ingenuity canonical pathway	BH^b^ *p*-value	Genes
Adipogenesis pathway	0.0022	*BMP4, BMPR1A, CLOCK, CTBP2, CTNNB1, EGR2, ERCC3,FGF1,FGFR2,FOXC2,FZD2,GTF2H1,GTF2H2,HAT1,HDAC1,HDAC4,HDAC6,HDAC7,HIF1A,KLF5,NFATC4,NR1D2,NR2F2,PPIP5K1,SAP30,SETDB1,SIRT1,SREBF1,TBL1XR1,TGFB1,TNF,TNFRSF1A,TP53,TXNIP,XBP1*
Aryl hydrocarbon receptor signaling	0.0044	*ALDH1A3,ALDH1L1,ALDH3A2,ALDH4A1,ALDH6A1,ALDH7A1,CDKN1A,FAS,GSTA1,GSTA2,GSTK1,GSTP1,HSPB2,HSPB7,IL6,MAPK3,MAPK8,MCM7,MDM2,MGST1,MGST3,NCOA7,NCOR2,NFIX,NQO2,NR2F1,RARB,RBL2,SMARCA4,TFF1,TGFB1,TGFB2,TNF,TP53*
PXR/RXR activation	0.0044	*ABCC2,ABCC3,AKT2,ALDH3A2,CES2,CES3,CYP3A5,CYP3A7,G6PC,GSTA1,GSTA2,HNF4A,IL6,NCOA1,NR1I3,PCK2,PRKACB,TNF,UGT1A1,UGT1A7*
Protein ubiquitination pathway	0.0044	*ANAPC2,ANAPC4,ANAPC5,BIRC2,BIRC6,CRYAA/CRYAA2,CRYAB,DNAJC10,DNAJC12,DNAJC22,DNAJC30,DNAJC7,HLA-A,HLA-B,HLA-C,HLA-E,HSPA2,HSPA4,HSPB2,HSPB7,HSPD1,MDM2,PSMA1,PSMA3,PSMB3,PSMB8,PSMC3,PSMD2,PSME2,SKP1,SKP2,SMURF2,TAP1,UBB,UBE2A,UBE2E1,UBE2E3,UBE2F,UBE2J2,UBE2Q2,UBR2,UCHL1,USP15,USP19,USP2,USP21,USP24,USP28,USP33,USP36,USP4,USP48,USP53,USP54,USP8*
Xenobiotic metabolism signaling	0.0044	*ABCC2,ABCC3,ALDH1A3,ALDH1L1,ALDH3A2,ALDH4A1,ALDH6A1,ALDH7A1,ANKRA2,CAMK1G,CAT,CES1,CES2,CES3,CHST1,CHST15,CITED2,CYP3A5,CYP3A7,DNAJC7,EIF2AK3,FMO1,FMO5,GSTA1,GSTA2,GSTK1,GSTP1,HDAC4,HS3ST6,IL6,MAF,MAOA,MAPK14,MAPK3,MAPK8,MAPK9,MGST1,MGST3,NCOA1,NCOR2,NDST2,NQO2,NR1I3,PIK3R4,PPP2R2B,PRKCB,PRKD3,PTPA,RAF1,SULT1C2,SULT2B1,TNF,UGT1A1,UGT1A6,UGT1A7,UGT2B7,UGT8,UST*
Apelin adipocyte signaling pathway	0.0044	*ADCY3,ADCY5,ADCY6,ADCY7,CAT,CYBB,GNAI2,GPX3,GPX8,GSTA1,GSTK1,GSTP1,HIF1A,MAPK14,MAPK15,MAPK3,MAPK8,MAPK9,MGST1,MGST3,NOX4,PRKAB1,PRKACB*
Death receptor signaling	0.0085	*ACTB,BIRC2,CASP2,CASP6,CASP7,FAS,HSPB2,HSPB7,IKBKG,LIMK1,MAPK8,PARP11,PARP12,PARP14,PARP6,PARP8,RIPK1,SPTAN1,TANK,TNF,TNFRSF10B,TNFRSF1A,TNFRSF25,TNFSF10*
LPS/IL-1-mediated inhibition of RXR function	0.0135	*ABCC2,ABCC3,ABCG1,ACOX2,ACSL4,ALDH1A3,ALDH1L1,ALDH3A2,ALDH4A1,ALDH6A1,ALDH7A1,APOE,CAT,CES2,CHST1,CHST15,CPT1B,CYP3A5,CYP3A7,CYP4A11,FABP1,FMO1,FMO5,GSTA1,GSTA2,GSTK1,GSTP1,HMGCS1,HS3ST6,IL1RL1,MAOA,MAPK8,MAPK9,MGST1,MGST3,NCOA1,NDST2,NR1I3,SLC27A2,SREBF1,SULT1C2,SULT2B1,TNF,TNFRSF1A,UST*
Hepatic fibrosis/hepatic stellate cell activation	0.0135	*AGTR1,CCL5,COL12A1,COL15A1,COL16A1,COL18A1,COL1A2,COL3A1,COL4A3,COL4A5,COL5A2,COL5A3,COL6A1,COL6A2,COL6A3,COL7A1,CSF1,ECE1,EDNRB,FAS,FGF1,FGFR2,FN1,IGF2,IL1RL1,IL6,IL6R,KLF6,LEPR,MYL6B,PDGFA,PDGFRB,SMAD4,TGFA,TGFB1,TGFB2,TIMP1,TNF,TNFRSF1A*
TR/RXR activation	0.0135	*ACACA,AKR1C1/AKR1C2,AKT2,BCL3,COL6A3,DIO1,G6PC,GPS2,HIF1A,LDLR,MDM2,NCOA1,NCOR2,PCK1,PIK3R4,SLC16A2,SLC16A3,SREBF1,SREBF2,TBL1XR1,THRA,UCP2*

^a^IPA, ingenuity pathway analysis; ^b^BH, Benjamini-Hochberg; LPS, lipopolysaccharide; IL-1, interleukin-1; PXR, pregnane X receptor; RXR, retinoic X receptor; TR, thyroid hormone receptor

The genes dysregulated by reperfusion (Cluster 3 and 4) were mainly related to cellular functions of growth, proliferation, and migration (semaphorin signaling, mTOR signaling, E74-like factor 2 [elF2] signaling, integrin signaling, apelin muscle signaling, and actin-based motility by Rho-related signaling) (Table 4). Semaphorins, a family of growth cone guidance molecules during neurodevelopment, interact with the members of the Rho family [33]. In particular, semaphorins can be synthesized in podocytes and tubular epithelial cells within the kidney and are implicated in cell migration, growth, and immune response in AKI [34, 35]. Integrin and actin signaling pathways linked to Rho signaling related to cytoskeletal remodeling process during cell growth and wound healing were enriched in the reperfusion effect clusters. In addition, immune response-related pathways such as antigen presentation pathway and sphingosine-1-phosphate signaling as well as intracellular and second messenger pathways were enriched.

**Table 4:** Top 10 most significantly enriched pathways in Cluster 3 and 4 using IPA^a^

Ingenuity canonical pathway	BH^b^ *p*-value	Genes
Semaphorin signaling in neurons	0.0003	*CFL1,FYN,LIMK2,MET,PAK4,PLXNB1,PTK2,RAC1,RHOBTB1,RHOC,RHOT2,RHOV,RND1,RND3,ROCK1,ROCK2,SEMA4D*
mTOR signaling	0.0003	*AKT1,AKT2,AKT3,EIF3B,EIF3D,EIF3E,EIF3F,EIF3H,EIF3J,EIF4A2,FKBP1A,IRS1,KRAS,MAPKAP1,PGF,PLD2,PPP2R2B,PPP2R5C,PRKAG1,PRR5,RAC1,RHOBTB1,RHOC,RHOT2,RHOV,RND1,RND3,RPS10,RPS13,RPS18,RPS2,RPS20,RPS24,RPS5,RPS6KA3,RPS8,RPSA*
eIF2 signaling	0.0003	*ACTB,AKT1,AKT2,AKT3,CDK11A,EIF2B1,EIF2B4,EIF3B,EIF3D,EIF3E,EIF3F,EIF3H,EIF3J,EIF4A2,HNRNPA1,KRAS,MYCN,PPP1CA,RPL12,RPL13A,RPL17,RPL24,RPL30,RPL31,RPL35A,RPL7,RPL8,RPS10,RPS13,RPS18,RPS2,RPS20,RPS24,RPS5,RPS8,RPSA,SREBF1,TRIB3*
Superpathway of cholesterol biosynthesis	0.0023	*ACAT1,HADHB,HMGCR,HSD17B7,LBR,MVD,MVK,SC5D,SQLE,TM7SF2*
Integrin signaling	0.0023	*ACTB,AKT1,AKT2,AKT3,ARF1,ARF4,ARPC2,ARPC3,ARPC5,ARPC5L,BCAR1,BCAR3,CAPN1,CAPN10,FYN,GRB7,ILK,KRAS,NEDD9,PAK4,PTK2,RAC1,RHOBTB1,RHOC,RHOT2,RHOV,RND1,RND3,ROCK1,TLN1,TNK2,TSPAN1,TSPAN4,ZYX*
Ephrin receptor signaling	0.0023	*ACP1,AKT1,AKT2,AKT3,ARHGEF15,ARPC2,ARPC3,ARPC5,ARPC5L,BCAR1,CFL1,CREBBP,EFNA1,FGF1,FYN,GNAS,GRINA,KRAS,LIMK2,PAK4,PDGFA,PGF,PTK2,PTPN13,RAC1,ROCK1,ROCK2,SDC2,SH2D3C,STAT3*
Apelin muscle signaling pathway	0.0023	*AKT1,AKT2,AKT3,APLNR,GNAS,NOS3,NRF1,PRKAG1*
Antigen presentation pathway	0.0055	*CALR,CD74,HLA-A,HLA-DMB,HLA-DOA,HLA-DQB2,HLA-F,MR1,NLRC5,TAP1,TAPBP*
Shingosine-1-phosphate signaling	0.0071	*ADCY3,ADCY4,AKT1,AKT2,AKT3,ASAH2B,CASP9,PDGFA,PDGFRB,PLCB1,PLCB4,PLCD1,PTK2,RAC1,RHOBTB1,RHOC,RHOT2,RHOV,RND1,RND3,S1PR1*
Regulation of actin-based motility by Rho	0.0072	*ACTB,ARHGDIA,ARPC2,ARPC3,ARPC5,ARPC5L,CFL1,PAK4,PIP5K1A,PIP5K1B,RAC1,RHOBTB1,RHOC,RHOT2,RHOV,RND1,RND3,ROCK1*

^a^IPA: ingenuity pathway analysis; ^b^BH, Benjamini-Hochberg; mTOR, mammalian target of rapamycin; Eif2, eukaryotic initiation factor 2

## Discussion

Although IRI is a critical factor to induce severe AKI and ESKD, the underlying biological mechanism is not well-established owing to the complexity of this pathophysiological process. Most previous studies have reported the molecular mechanism of IRI as a single process without separately evaluating the consequences of ischemia and reperfusion. Therefore, here we evaluated the biological signatures related to each event during IRI at the transcriptomic level in human kidney samples using RNA-seq.

We performed differential expression analysis and applied machine learning to identify the key genes and pathways affected during IRI. We compared pre-ischemia and ischemia (ischemia versus pre-ischemia), ischemia and reperfusion (reperfusion versus ischemia), and pre-ischemia and reperfusion (reperfusion versus pre-ischemia) conditions. While the conventional differential expression analysis process only compared two conditions, we applied unbiased clustering algorithm, SOM, to identify specific trajectories of interest across all three conditions. In particular, we focused on specific gene expression patterns to identify the effects of ischemic and reperfusion separately during IRI. (1) The genes dysregulated in ischemia versus pre-ischemia; (2) those dysregulated in reperfusion versus ischemia. We then performed pathway analysis to investigate the affected pathways in ischemia and reperfusion during IRI process using selected genes from differential expression analysis and SOM clustering.

Pathway analysis for DEGs in each comparison group revealed the enrichment of metabolism-related pathways in ischemia. On the other hand, cellular assembly, development and migration, and immune response-related pathways were enriched in reperfusion. Pathway analysis for the genes selected from SOM revealed apoptosis, xenobiotic metabolism, and fibrosis-related pathways to be enriched in ischemia and cell growth and migration and immune response-related pathways to be significantly enriched in reperfusion.

In general, the interruption of the blood supply during ischemia induces changes in specific metabolic pathways. We found that melatonin/serotonin degradation and FXR/RXR pathway related to lipid metabolism as well as bupropion and acetone degradation, estrogen biosynthesis, and PXR/RXR pathway related to drug metabolism were enriched in ischemia. Melatonin and its metabolites have been regarded as scavengers of free radicals or stimulators of antioxidant enzymes, and play protective roles in kidney ischemic injury [36]. Thus, certain metabolites associated with early ischemic damage may be used as pathogenic biomarkers. Therefore, further investigations are warranted to understand the role of these metabolites in ischemia. Further, we observed that the pathways related to apoptosis, fibrosis, and adipogenesis were significantly enriched by ischemia; pro-apoptotic genes such as *FAS*, *CAS2/6/7*, *PARP6/8/11/12/14*, *TNF*, and *TNFRSF1/10/10B/25*, fibrosis-related genes, collagen (*COL1/3/ 4/5/6/7/12//15/16/18*), and adipogenesis-related genes were downregulated, suggesting the existence of a protective process from cell damage against ischemia injury in the kidney tissue.

Reperfusion after ischemia triggers a robust inflammatory response within the kidney by blood re-supply. The immune response-related pathways, including inflammasome and antigen presentation pathway, were dysregulated after reperfusion in our study, and this effect was not observed during ischemia. Considering the other mechanisms of reperfusion, cell development, growth and migration-related pathways such as FGF, mammalian target of rapamycin (mTOR), eukaryotic initiation factor 2 (eIF2), semaphorin, integrin, and actin-based motility by Rho signaling were identified. *AKT1/2/3*, *KRAS*, *MAPKAP1*, *EIF2B1/4*, *CDK11A*, *RPL8/12/13A/17/24/30/31*, *ARPC2/3/5/5 L*, *RAC1,* and *RHOC/T2/V*, crucial for regeneration and repair system after reperfusion injury were downregulated. Thus, it indicates that a dysregulation in cell growth and migration pathways in response to reperfusion occurs.

Semaphorin 4D (*SEMA4D*) and plexin B1 (*PLXNB1*), a receptor of semaphorin related to semaphorin signaling, were found to be downregulated under reperfusion condition. Semaphorin 3A (Sema3A) promotes kidney injury followed by AKI [37]; however, the role of Sema4D is not well studied. Previous preclinical and clinical studies have shown that Sema3A is detectable in urine, suggestive of its potential role as a biomarker of AKI [38–40]. In comparison with Sema3A, Sema4D is a transmembrane protein and an insoluble factor. Thus, further investigations are needed to evaluate the role of Sema4D and plexin B1 as potential biomarkers of IRI.

## Conclusion

We reveal that specific biological pathways were uniquely involved in ischemia and reperfusion during IRI. Metabolism, apoptosis, and fibrosis-related pathways were significantly dysregulated under ischemia conditions, whereas cell development, growth, migration, and immune response-related pathways were affected by reperfusion following ischemia. Therefore, we suggest that a condition-specific therapeutic strategy may be necessary to prevent severe kidney damage after IRI in the clinical setting. Although our study has limitations such as the small number of samples and relatively short duration of ischemia and reperfusion, we believe that it will contribute to the understanding of the mechanisms underlying IRI.

## Supplementary information

**Additional file 1: Supplementary Table S1.** Clinical characteristics of patients. **Supplementary Table S2.** Information of RNA sequencing analysis. **Supplementary Table S3.** Top 20 most significantly DEGs between ischemia vs. pre-ischemia. **Supplementary Table S4.** Top 20 most significantly DEGs between reperfusion vs. ischemia. **Supplementary Table S5.** Top 20 most significantly DEGs between reperfusion vs. pre-ischemia. **Supplementary Table S6.** Pathways for DEGs between ischemia and pre-ischemia using IPA. **Supplementary Table S7.** Pathways for DEGs between reperfusion and ischemia using IPA. **Supplementary Table S8.** Pathways for DEGs between reperfusion and pre-ischemia using IPA.

## Data Availability

RNA-seq data are available in Gene Expression Omnibus (GEO) database under the accession number GSE142077.

## Abbreviations

AKI: Acute kidney injury
IRI: Ischemia-reperfusion injury
ESKD: End-stage kidney disease
SOM: Self-organizing map
KDIGO: Kidney Disease: Improving Global Outcomes
CKD: Chronic kidney disease
RNA-seq: RNA sequencing
eGFR: Estimated glomerular filtration rate
qPCR: Quantitative polymerase chain reaction
GEO: Gene Expression Omnibus
DEGs: Differentially expressed genes
IPA: Ingenuity Pathway Analysis

## References

[1] Lameire N, Van Massenhove J, Van Biesen W (2012). What is the difference between prerenal and renal acute kidney injury?. Acta Clin Belg.

[2] Lameire NH, Bagga A, Cruz D, De Maeseneer J, Endre Z, Kellum JA, Liu KD, Mehta RL, Pannu N, Van Biesen W (2013). Acute kidney injury: an increasing global concern. Lancet.

[3] Kundert F, Platen L, Iwakura T, Zhao Z, Marschner JA, Anders HJ (2018). Immune mechanisms in the different phases of acute tubular necrosis. Kidney Res Clin Pract.

[4] Susantitaphong P, Cruz DN, Cerda J, Abulfaraj M, Alqahtani F, Koulouridis I, Jaber BL, Acute kidney injury advisory Group of the American Society of N (2013). World incidence of AKI: a meta-analysis. Clin J Am Soc Nephrol.

[5] Park S, Lee S, Jo HA, Han K, Kim Y, An JN, Joo KW, Lim CS, Kim YS, Kim H (2018). Epidemiology of continuous renal replacement therapy in Korea: results from the National Health Insurance Service claims database from 2005 to 2016. Kidney Res Clin Pract.

[6] Chawla LS, Kimmel PL (2012). Acute kidney injury and chronic kidney disease: an integrated clinical syndrome. Kidney Int.

[7] Chawla LS, Eggers PW, Star RA, Kimmel PL (2014). Acute kidney injury and chronic kidney disease as interconnected syndromes. N Engl J Med.

[8] Dorweiler B, Pruefer D, Andrasi TB, Maksan SM, Schmiedt W, Neufang A, Vahl CF (2007). Ischemia-reperfusion injury : pathophysiology and clinical implications. Eur J Trauma Emerg Surg.

[9] Murugan R, Kellum JA (2011). Acute kidney injury: what's the prognosis?. Nat Rev Nephrol.

[10] Soranno DE, Gil HW, Kirkbride-Romeo L, Altmann C, Montford JR, Yang H, Levine A, Buchanan J, Faubel S (2019). Matching human unilateral AKI, a reverse translational approach to investigate kidney recovery after ischemia. J Am Soc Nephrol.

[11] Huang H, van Dullemen LFA, Akhtar MZ, Faro ML, Yu Z, Valli A, Dona A, Thezenas ML, Charles PD, Fischer R (2018). Proteo-metabolomics reveals compensation between ischemic and non-injured contralateral kidneys after reperfusion. Sci Rep.

[12] Menne J, Dumann E, Haller H, Schmidt BMW (2019). Acute kidney injury and adverse renal events in patients receiving SGLT2-inhibitors: a systematic review and meta-analysis. PLoS Med.

[13] Okusa MD, Rosner MH, Kellum JA, Ronco C, Acute Dialysis quality initiative XW (2016). Therapeutic targets of human AKI: harmonizing human and animal AKI. J Am Soc Nephrol.

[14] van der Worp HB, Howells DW, Sena ES, Porritt MJ, Rewell S, O'Collins V, Macleod MR (2010). Can animal models of disease reliably inform human studies?. PLoS Med.

[15] Kohonen T (2013). Essentials of the self-organizing map. Neural Netw.

[16] Cippa PE, Sun B, Liu J, Chen L, Naesens M, McMahon AP. Transcriptional trajectories of human kidney injury progression. JCI Insight. 2018;3(22). 10.1172/jci.insight.123151 PMC6302941 30429361

[17] Patro R, Duggal G, Love MI, Irizarry RA, Kingsford C (2017). Salmon provides fast and bias-aware quantification of transcript expression. Nat Methods.

[18] Soneson C, Love MI, Robinson MD (2015). Differential analyses for RNA-seq: transcript-level estimates improve gene-level inferences. F1000Res.

[19] Love MI, Huber W, Anders S (2014). Moderated estimation of fold change and dispersion for RNA-seq data with DESeq2. Genome Biol.

[20] Nebert DW, Russell DW (2002). Clinical importance of the cytochromes P450. Lancet.

[21] Tukey RH, Strassburg CP (2000). Human UDP-glucuronosyltransferases: metabolism, expression, and disease. Annu Rev Pharmacol Toxicol.

[22] Tarocco A, Caroccia N, Morciano G, Wieckowski MR, Ancora G, Garani G, Pinton P (2019). Melatonin as a master regulator of cell death and inflammation: molecular mechanisms and clinical implications for newborn care. Cell Death Dis.

[23] Oladimeji PO, Chen T (2018). PXR: more than just a master xenobiotic receptor. Mol Pharmacol.

[24] Itoh N, Ohta H (2013). Roles of FGF20 in dopaminergic neurons and Parkinson's disease. Front Mol Neurosci.

[25] Strutz F, Zeisberg M, Ziyadeh FN, Yang CQ, Kalluri R, Muller GA, Neilson EG (2002). Role of basic fibroblast growth factor-2 in epithelial-mesenchymal transformation. Kidney Int.

[26] Ramaswamy S, Tamayo P, Rifkin R, Mukherjee S, Yeang CH, Angelo M, Ladd C, Reich M, Latulippe E, Mesirov JP (2001). Multiclass cancer diagnosis using tumor gene expression signatures. Proc Natl Acad Sci U S A.

[27] Covell DG, Wallqvist A, Rabow AA, Thanki N (2003). Molecular classification of cancer: unsupervised self-organizing map analysis of gene expression microarray data. Mol Cancer Ther.

[28] Hinder LM, Park M, Rumora AE, Hur J, Eichinger F, Pennathur S, Kretzler M, Brosius FC, Feldman EL (2017). Comparative RNA-Seq transcriptome analyses reveal distinct metabolic pathways in diabetic nerve and kidney disease. J Cell Mol Med.

[29] Hinder LM, Murdock BJ, Park M, Bender DE, O'Brien PD, Rumora AE, Hur J, Feldman EL (2018). Transcriptional networks of progressive diabetic peripheral neuropathy in the db/db mouse model of type 2 diabetes: An inflammatory story. Exp Neurol.

[30] Tamayo P, Slonim D, Mesirov J, Zhu Q, Kitareewan S, Dmitrovsky E, Lander ES, Golub TR (1999). Interpreting patterns of gene expression with self-organizing maps: methods and application to hematopoietic differentiation. Proc Natl Acad Sci U S A.

[31] Toronen P, Kolehmainen M, Wong G, Castren E (1999). Analysis of gene expression data using self-organizing maps. FEBS Lett.

[32] Li B, Ruotti V, Stewart RM, Thomson JA, Dewey CN (2010). RNA-Seq gene expression estimation with read mapping uncertainty. Bioinformatics.

[33] Siebold C, Jones EY (2013). Structural insights into semaphorins and their receptors. Semin Cell Dev Biol.

[34] Xia J, Swiercz JM, Banon-Rodriguez I, Matkovic I, Federico G, Sun T, Franz T, Brakebusch CH, Kumanogoh A, Friedel RH (2015). Semaphorin-Plexin signaling controls mitotic spindle orientation during epithelial morphogenesis and repair. Dev Cell.

[35] Xia J, Worzfeld T (2016). Semaphorins and Plexins in kidney disease. Nephron.

[36] Shi S, Lei S, Tang C, Wang K, Xia Z. Melatonin attenuates acute kidney ischemia/reperfusion injury in diabetic rats by activation of the SIRT1/Nrf2/HO-1 signaling pathway. Biosci Rep. 2019;39(1):BSR20181614. 10.1042/BSR20181614 PMC6331666 30578379

[37] Song MF, Yang Y, Yi ZW, Zhang ZQ, Shen XD, Hu GH, Zhu YF (2018). Sema 3A as a biomarker of the activated mTOR pathway during hexavalent chromium-induced acute kidney injury. Toxicol Lett.

[38] Jayakumar C, Ranganathan P, Devarajan P, Krawczeski CD, Looney S, Ramesh G (2013). Semaphorin 3A is a new early diagnostic biomarker of experimental and pediatric acute kidney injury. PLoS One.

[39] Doi K, Noiri E, Nangaku M, Yahagi N, Jayakumar C, Ramesh G (2014). Repulsive guidance cue semaphorin 3A in urine predicts the progression of acute kidney injury in adult patients from a mixed intensive care unit. Nephrol Dial Transplant.

[40] Lewandowska L, Matuszkiewicz-Rowinska J, Jayakumar C, Oldakowska-Jedynak U, Looney S, Galas M, Dutkiewicz M, Krawczyk M, Ramesh G (2014). Netrin-1 and semaphorin 3A predict the development of acute kidney injury in liver transplant patients. PLoS One.

