# Supplementary material for: RNA-Seq identifies condition-specific biological signatures of ischemia-reperfusion injury in the human kidney
Source: BMC Nephrol. 2020 Sep 25;21(Suppl 1):398. doi: 10.1186/s12882-020-02025-y (PMC7517631; doi:10.1186/s12882-020-02025-y)
Supplement: Supplementary file 1 — Additional file 1: Supplementary Table S1. Clinical characteristics of patients. Supplementary Table S2. Information of RNA sequencing analysis. Supplementary Table S3. Top 20 most significantly DEGs between ischemia vs. pre-ischemia. Supplementary Table S4. Top 20 most significantly DEGs between reperfusion vs. ischemia. Supplementary Table S5. Top 20 most significantly DEGs between reperfusion vs. pre-ischemia. Supplementary Table S6. Pathways for DEGs between ischemia and pre-ischemia using IPA. Supplementary Table S7. Pathways for DEGs between reperfusion and ischemia using IPA. Supplementary Table S8. Pathways for DEGs between reperfusion and pre-ischemia using IPA. [file 12882_2020_2025_MOESM1_ESM.docx]

Supplementary Tables

RNA-Seq identifies condition-specific biological signatures of ischemia-reperfusion injury in the human kidney

Meeyoung Park^†^

Biomedical Research Institute, Pusan National University Hospital, Busan, South Korea, mpark.pnuh@gmail.com

Chae Hwa Kwon^†^

Biomedical Research Institute, Pusan National University Hospital, Busan, South Korea, [chkwon@pusan.ac.kr](mailto:chkwon@pusan.ac.kr)

Hong Koo Ha

Department of Urology, Pusan National University Hospital, Busan, South Korea, hongkooha@pusan.ac.kr

Miyeun Han

Department of Internal Medicine, Pusan National University Hospital, Busan, South Korea, myeun81@hanmail.net

Sang Heon Song^*^

Department of Internal Medicine and Biomedical Research Institute, Pusan National University Hospital, Busan, South Korea, shsong0209@gmail.com

^†^ These authors contributed equally. ^*^ Corresponding author.

Supplementary Table S1. Clinical characteristics of patients

| Patients  (number) | Age  (years) | Sex | Creatinine  (mg/dL) | eGFR  (ml/min/1.73m2) | CKD (stage) | Hb  (g/dL) | Proteinuria |
| --- | --- | --- | --- | --- | --- | --- | --- |
| 1 | 73 | male | 0.91 | 81.7 | 2 | 14 | Negative |
| 2 | 50 | male | 1.06 | 74 | 2 | 14.8 | Negative |
| 3 | 66 | male | 0.76 | 102.9 | 1 | 13.2 | Negative |
| 4 | 66 | male | 0.94 | 80.3 | 2 | 16 | Negative |
| 5 | 69 | male | 0.76 | 101.7 | 1 | 13 | Negative |

eGFR, estimated glomerular filtration rate; CKD, chronic kidney disease; Hb, Hemoglobin

Supplementary Table S2. Information of RNA sequencing analysis

| Sample name | Run format | Max Read Length | Total Reads | Mapped Reads | Mapping Rates (%) |
| --- | --- | --- | --- | --- | --- |
| P-IRI-h-2 | 101X2 | 101 | 55,956,090 | 52,732,660 | 84.38 |
| I-IRI-h-2 | 101X2 | 101 | 53,906,202 | 50,792,594 | 80.52 |
| R10-IRI-h-2 | 101X2 | 101 | 56,877,848 | 53,726,528 | 82.15 |
| P-IRI-h-3 | 101X2 | 101 | 55,774,666 | 52,471,077 | 82.21 |
| I-IRI-h-3 | 101X2 | 101 | 51,925,412 | 49,130,670 | 81.47 |
| R10-IRI-h-3 | 101X2 | 101 | 58,632,636 | 55,249,877 | 82.41 |
| P-IRI-h-4 | 101X2 | 101 | 53,924,228 | 49,993,942 | 78.43 |
| I-IRI-h-4 | 101X2 | 101 | 55,217,432 | 51,325,239 | 77.26 |
| R10-IRI-h-4 | 101X2 | 101 | 56,178,044 | 52,252,233 | 78.04 |
| P-IRI-h-5 | 101X2 | 101 | 54,110,358 | 50,206,441 | 78.00 |
| I-IRI-h-5 | 101X2 | 101 | 75,877,790 | 71,082,042 | 83.93 |
| R10-IRI-h-5 | 101X2 | 101 | 73,729,052 | 69,526,194 | 83.84 |
| P-IRI-h-6 | 101X2 | 101 | 70,157,640 | 65,640,744 | 81.14 |
| R10-IRI-h-6 | 101X2 | 101 | 75,685,582 | 71,255,453 | 85.84 |
| I-IRI-h-6 | 101X2 | 101 | 68,619,310 | 64,648,603 | 83.63 |
| Average | 101X2 | 101 | 61,104,819 | 57,335,620 | 81.55 |

Supplementary Table S3. Top 20 most significantly DEGs between ischemia *vs*. pre-ischemia

| Gene symbol | Description | P-value |
| --- | --- | --- |
| COMMD3-BMI1 | COMMD3-BMI1 readthrough | 0.00012 |
| ADRA1D | adrenoceptor alpha 1D | 0.00014 |
| TNNI1 | troponin I1, slow skeletal type | 0.00023 |
| NKAIN4 | sodium/potassium transporting ATPase interacting 4 | 0.00024 |
| RP11_74C12 | RP11_74C12 | 0.00042 |
| TSPY1 | testis specific protein Y-linked 1 | 0.00048 |
| NXF3 | nuclear RNA export factor 3 | 0.00054 |
| LOC102724163 | uncharacterized LOC102724163 | 0.00066 |
| SLC7A14 | solute carrier family 7 member 14 | 0.00080 |
| RIMS4 | regulating synaptic membrane exocytosis 4 | 0.00088 |
| ARL2-SNX15 | ARL2-SNX15 readthrough (NMD candidate) | 0.00092 |
| SMOC1 | SPARC related modular calcium binding 1 | 0.00097 |
| REN | renin | 0.00118 |
| MROH2A | maestro heat like repeat family member 2A | 0.00125 |
| NELL2 | neural EGFL like 2 | 0.00131 |
| SSC5D | scavenger receptor cysteine rich family member with 5 domains | 0.00179 |
| GDF10 | growth differentiation factor 10 | 0.00180 |
| LOC100133920 | methylenetetrahydrofolate dehydrogenase (NADP+ dependent) 1 like pseudogene | 0.00209 |
| LINC00167 | long intergenic non-protein coding RNA 167 | 0.00225 |
| SIGLEC5 | sialic acid binding Ig like lectin 5 | 0.00237 |

Supplementary Table S4. Top 20 most significantly DEGs between reperfusion *vs*. ischemia

| Gene symbol | Description | P-value |
| --- | --- | --- |
| HCG9 | HLA complex group 9 | 0.00142 |
| ADIPOQ | adiponectin, C1Q and collagen domain containing | 0.00144 |
| NELL2 | neural EGFL like 2 | 0.00303 |
| BCL2L2-PABPN1 | BCL2L2-PABPN1 readthrough | 0.00344 |
| KCNA6 | potassium voltage-gated channel subfamily A member 6 | 0.00398 |
| INO80B-WBP1 | INO80B-WBP1 readthrough (NMD candidate) | 0.00647 |
| H2AC18 | H2A clustered histone 18 | 0.00692 |
| TRPM1 | transient receptor potential cation channel subfamily M member 1 | 0.00754 |
| ADRA1D | adrenoceptor alpha 1D | 0.00807 |
| DNAAF4-CCPG1 | DNAAF4-CCPG1 readthrough (NMD candidate) | 0.00836 |
| LOC101928626 | uncharacterized LOC101928626 | 0.01010 |
| GRIN3A | glutamate ionotropic receptor NMDA type subunit 3A | 0.01038 |
| TMEM265 | transmembrane protein 265 | 0.01120 |
| DLEU7 | deleted in lymphocytic leukemia 7 | 0.01148 |
| IL18RAP | interleukin 18 receptor accessory protein | 0.01218 |
| RNA5-8SN2 | RNA, 5.8S ribosomal N2 | 0.01397 |
| RECQL4 | RecQ like helicase 4 | 0.01503 |
| PLXNC1 | plexin C1 | 0.01514 |
| FGF22 | fibroblast growth factor 22 | 0.01530 |
| PRPH | peripherin | 0.01666 |

Supplementary Table S5. Top 20 most significantly DEGs between reperfusion *vs*. pre-ischemia

| Gene symbol | Description | P-value |
| --- | --- | --- |
| TNNI1 | troponin I1, slow skeletal type | 2.E-09 |
| ILDR2 | immunoglobulin like domain containing receptor 2 | 3.E-08 |
| DPP6 | dipeptidyl peptidase like 6 | 2.E-07 |
| NTRK1 | neurotrophic receptor tyrosine kinase 1 | 3.E-07 |
| WT1 | WT1 transcription factor | 1.E-06 |
| SLC16A1 | solute carrier family 16 member 1 | 5.E-06 |
| CLIC5 | chloride intracellular channel 5 | 5.E-06 |
| LMX1B | LIM homeobox transcription factor 1 beta | 7.E-06 |
| FGF1 | fibroblast growth factor 1 | 7.E-06 |
| IQSEC3 | IQ motif and Sec7 domain ArfGEF 3 | 9.E-06 |
| TENM2 | teneurin transmembrane protein 2 | 9.E-06 |
| FGF5 | fibroblast growth factor 5 | 2.E-05 |
| NTNG1 | netrin G1 | 2.E-05 |
| TYRO3 | TYRO3 protein tyrosine kinase | 2.E-05 |
| PDZD7 | PDZ domain containing 7 | 2.E-05 |
| PTPRO | protein tyrosine phosphatase receptor type O | 2.E-05 |
| REN | renin | 3.E-05 |
| PTPRQ | protein tyrosine phosphatase receptor type Q | 3.E-05 |
| DDX10 | DEAD-box helicase 10 | 4.E-05 |
| SCN9A | sodium voltage-gated channel alpha subunit 9 | 5.E-05 |

Supplementary Table S6. Pathways for DEGs between ischemia and pre-ischemia using IPA

| Ingenuity Canonical Pathways | P-value | Genes |
| --- | --- | --- |
| Nicotine Degradation III | < 0.00001 | CYP1A2,CYP2C8,CYP2C9,CYP2J2,CYP3A4,CYP3A7,UGT1A1,UGT1A6,UGT1A7,UGT2A3,UGT2B11,UGT3A1 |
| Melatonin Degradation I | < 0.00001 | CYP1A2,CYP2C8,CYP2C9,CYP2J2,CYP3A4,CYP3A7,UGT1A1,UGT1A6,UGT1A7,UGT2A3,UGT2B11,UGT3A1 |
| Nicotine Degradation II | < 0.00001 | CYP1A2,CYP2C8,CYP2C9,CYP2J2,CYP3A4,CYP3A7,UGT1A1,UGT1A6,UGT1A7,UGT2A3,UGT2B11,UGT3A1 |
| Superpathway of Melatonin Degradation | < 0.00001 | CYP1A2,CYP2C8,CYP2C9,CYP2J2,CYP3A4,CYP3A7,UGT1A1,UGT1A6,UGT1A7,UGT2A3,UGT2B11,UGT3A1 |
| Serotonin Degradation | < 0.00001 | ADH1B,AKR1A1,ALDH4A1,DHRS4,UGT1A1,UGT1A6,UGT1A7,UGT2A3,UGT2B11,UGT3A1 |
| Thyroid Hormone Metabolism II (via Conjugation and/or Degradation) | < 0.00001 | DIO2,DIO3,UGT1A1,UGT1A6,UGT1A7,UGT2A3,UGT2B11,UGT3A1 |
| PXR/RXR Activation | < 0.00001 | ABCC2,CYP1A2,CYP2C8,CYP2C9,CYP3A4,CYP3A7,PCK2,UGT1A1,UGT1A7 |
| Bupropion Degradation | < 0.00001 | CYP1A2,CYP2C8,CYP2C9,CYP2J2,CYP3A4,CYP3A7 |
| Acetone Degradation I (to Methylglyoxal) | 0.00001 | CYP1A2,CYP2C8,CYP2C9,CYP2J2,CYP3A4,CYP3A7 |
| Estrogen Biosynthesis | 0.00008 | CYP1A2,CYP2C8,CYP2C9,CYP2J2,CYP3A4,CYP3A7 |
| Xenobiotic Metabolism Signaling | 0.00065 | ABCC2,ALDH4A1,CES1,CYP1A2,CYP2C8,CYP2C9,CYP3A4,CYP3A7,NQO2,SULT4A1,UGT1A1,UGT1A6,UGT1A7,UGT2B11 |
| Thyronamine and Iodothyronamine Metabolism | 0.00093 | DIO2,DIO3 |
| Thyroid Hormone Metabolism I (via Deiodination) | 0.00093 | DIO2,DIO3 |
| Guanosine Nucleotides Degradation III | 0.00107 | GDA,NT5C1A,NT5E |
| Ethanol Degradation II | 0.00234 | ADH1B,AKR1A1,ALDH4A1,DHRS4 |
| Tyrosine Degradation I | 0.00302 | FAH,HGD |
| Noradrenaline and Adrenaline Degradation | 0.00331 | ADH1B,AKR1A1,ALDH4A1,DHRS4 |
| Purine Nucleotides Degradation II (Aerobic) | 0.00372 | GDA,NT5C1A,NT5E |
| Phospholipases | 0.00513 | NOTUM,PLA2G7,PLAAT2,PLCZ1,PNPLA3 |
| FXR/RXR Activation | 0.00724 | ABCC2,C4A/C4B,FBP1,ITIH4,PCK2,SAA2,SLC51B |
| Bladder Cancer Signaling | 0.00759 | FGF1,FGF10,FGF5,FGF7,MMP24,MMP9 |
| Antioxidant Action of Vitamin C | 0.01318 | NOTUM,PLA2G7,PLAAT2,PLCZ1,PNPLA3,SLC23A3 |
| Granulocyte Adhesion and Diapedesis | 0.01479 | CCL11,CCL13,CCL15,CCL26,CSF3,IL1RL1,MMP24,MMP9 |
| FGF Signaling | 0.01698 | CREB3L3,FGF1,FGF10,FGF5,FGF7 |
| Neuroprotective Role of THOP1 in Alzheimer's Disease | 0.01738 | ACE,CFD,KLK6,KLK7,MME,MMP9 |
| Sorbitol Degradation I | 0.01778 | SORD |
| LPS/IL-1 Mediated Inhibition of RXR Function | 0.01905 | ABCC2,ALDH4A1,CYP2C8,CYP2C9,CYP3A4,CYP3A7,FABP4,IL1RL1,SULT4A1 |
| Acyl-CoA Hydrolysis | 0.02138 | ACOT4,ACOT7 |
| Urate Biosynthesis/Inosine 5'-phosphate Degradation | 0.02138 | NT5C1A,NT5E |
| Gap Junction Signaling | 0.02512 | ACTG2,GJA3,GJB1,GJB2,GJC3,HTR2A,NOTUM,PLCZ1 |
| Complement System | 0.02754 | C4A/C4B,CFD,CFI |
| Adenosine Nucleotides Degradation II | 0.02818 | NT5C1A,NT5E |
| Inhibition of Matrix Metalloproteases | 0.03162 | MMP24,MMP9,THBS2 |
| 4-hydroxyproline Degradation I | 0.03548 | ALDH4A1 |
| Retinol Biosynthesis | 0.03802 | CES1,PNPLA3,RBP5 |
| GPCR-Mediated Integration of Enteroendocrine Signaling Exemplified by an L Cell | 0.04074 | GAL,NOTUM,PLCZ1,PYY |
| PFKFB4 Signaling Pathway | 0.04786 | CREB3L3,FBP1,GCK |
| VDR/RXR Activation | 0.05012 | IL1RL1,KLK6,TRPV5,WT1 |

Supplementary Table S7. Pathways for DEGs between reperfusion and ischemia using IPA

| Ingenuity Canonical Pathways | P-value | Genes |
| --- | --- | --- |
| FGF Signaling | 0.00093 | FGF22,FGF5,FGF7 |
| Clathrin-mediated Endocytosis Signaling | 0.001 | FGF22,FGF5,FGF7,TF |
| Bladder Cancer Signaling | 0.00145 | FGF22,FGF5,FGF7 |
| Cardiac Hypertrophy Signaling (Enhanced) | 0.00501 | ADRA1D,FGF22,FGF5,FGF7,IL18RAP |
| Regulation of the Epithelial-Mesenchymal Transition Pathway | 0.00977 | FGF22,FGF5,FGF7 |
| AMPK Signaling | 0.01259 | ADIPOQ,ADRA1D,CHRNB4 |
| Actin Cytoskeleton Signaling | 0.01349 | FGF22,FGF5,FGF7 |
| Amyotrophic Lateral Sclerosis Signaling | 0.02089 | GRIN3A,PRPH |
| LXR/RXR Activation | 0.03162 | IL18RAP,TF |
| Synaptogenesis Signaling Pathway | 0.03467 | CDH8,GRIN3A,SNCG |
| Inflammasome pathway | 0.04467 | NLRC4 |

Supplementary Table S8. Pathways for DEGs between reperfusion and pre-ischemia using IPA

| Ingenuity Canonical Pathways | P-value | Genes |
| --- | --- | --- |
| Nicotine Degradation II | < 0.00001 | AOX1,CYP1A1,CYP2B6,CYP2C8,CYP2C9,CYP2J2,CYP3A4,CYP3A5,CYP3A7,FMO3,FMO4,UGT1A1,UGT1A4,UGT1A6,UGT1A7,UGT2A3,UGT2B7,UGT3A1 |
| FXR/RXR Activation | < 0.00001 | ABCC2,AGT,ALB,APOA1,APOC3,APOE,APOH,APOM,C4A/C4B,CYP27A1,CYP8B1,FBP1,FGFR4,HNF1A,HNF4A,ITIH4,MLXIPL,MTTP,PCK2,SERPINF2,SLC10A2,SLC51A,SLC51B,VTN |
| Nicotine Degradation III | < 0.00001 | AOX1,CYP1A1,CYP2B6,CYP2C8,CYP2C9,CYP2J2,CYP3A4,CYP3A5,CYP3A7,UGT1A1,UGT1A4,UGT1A6,UGT1A7,UGT2A3,UGT2B7,UGT3A1 |
| Serotonin Degradation | < 0.00001 | ADH1B,ADH6,AKR1A1,ALDH1B1,ALDH2,ALDH3B1,ALDH4A1,ALDH7A1,DHRS4,UGT1A1,UGT1A4,UGT1A6,UGT1A7,UGT2A3,UGT2B7,UGT3A1 |
| Xenobiotic Metabolism Signaling | < 0.00001 | ABCC2,ALDH1B1,ALDH2,ALDH3B1,ALDH4A1,ALDH6A1,ALDH7A1,ALDH8A1,CAMK2A,CES1,CES2,CES3,CHST13,CYP1A1,CYP2B6,CYP2C8,CYP2C9,CYP3A4,CYP3A5,CYP3A7,FMO3,FMO4,FTL,GSTA1,HMOX1,HS3ST6,MAF,MGST1,NQO2,UGT1A1,UGT1A4,UGT1A6,UGT1A7,UGT2B7 |
| Melatonin Degradation I | < 0.00001 | CYP1A1,CYP2B6,CYP2C8,CYP2C9,CYP2J2,CYP3A4,CYP3A5,CYP3A7,UGT1A1,UGT1A4,UGT1A6,UGT1A7 ,UGT2A3,UGT2B7,UGT3A1 |
| PXR/RXR Activation | < 0.00001 | ABCC2,CES2,CES3,CYP2B6,CYP2C8,CYP2C9,CYP3A4,CYP3A5,CYP3A7,GSTA1,HNF4A,IGFBP1,PCK2,UGT1A1,UGT1A7 |
| Superpathway of Melatonin  Degradation | < 0.00001 | CYP1A1,CYP2B6,CYP2C8,CYP2C9,CYP2J2,CYP3A4,CYP3A5,CYP3A7,UGT1A1,UGT1A4,UGT1A6,UGT1A7 ,UGT2A3,UGT2B7,UGT3A1 |
| LPS/IL-1 Mediated Inhibition of RXR Function | < 0.00001 | ABCC2,ACOX2,ACSBG2,ALDH1B1,ALDH2,ALDH3B1,ALDH4A1,ALDH6A1,ALDH7A1,ALDH8A1,APOE,CES2,CHST13,CYP2B6,CYP2C8,CYP2C9,CYP3A4,CYP3A5,CYP3A7,FABP1,FMO3,FMO4,GSTA1,HS3ST6,IL1RL1,MGST1,SLC27A2 |
| Ethanol Degradation II | < 0.00001 | ACSS2,ADH1B,ADH6,AKR1A1,ALDH1B1,ALDH2,ALDH3B1,ALDH4A1,ALDH7A1,DHRS4 |
| Glycine Betaine Degradation | < 0.00001 | BHMT,BHMT2,DMGDH,PIPOX,SARDH,SHMT1 |
| Acetone Degradation I  (to Methylglyoxal) | < 0.00001 | CYP1A1,CYP2B6,CYP2C8,CYP2C9,CYP2J2,CYP3A4,CYP3A5,CYP3A7,CYP4A22 |
| Estrogen Biosynthesis | < 0.00001 | AKR1C3,CYP1A1,CYP2B6,CYP2C8,CYP2C9,CYP2J2,CYP3A4,CYP3A5,CYP3A7,HSD17B14 |
| Bupropion Degradation | < 0.00001 | CYP1A1,CYP2B6,CYP2C8,CYP2C9,CYP2J2,CYP3A4,CYP3A5,CYP3A7 |
| Bile Acid Biosynthesis, Neutral  Pathway | < 0.00001 | AKR1C1/AKR1C2,AKR1C3,AMACR,CYP27A1,CYP3A4,CYP8B1 |
| Noradrenaline and Adrenaline  Degradation | < 0.00001 | ADH1B,ADH6,AKR1A1,ALDH1B1,ALDH2,ALDH3B1,ALDH4A1,ALDH7A1,DHRS4 |
| Tryptophan Degradation X (Mammalian, via Tryptamine) | 0.00003 | AKR1A1,ALDH1B1,ALDH2,ALDH3B1,ALDH4A1,ALDH7A1,DDC |
| Valine Degradation I | 0.00004 | ABAT,ACADSB,ALDH6A1,ECHS1,EHHADH,HIBCH |
| Oxidative Ethanol Degradation III | 0.00005 | ACSS2,ALDH1B1,ALDH2,ALDH3B1,ALDH4A1,ALDH7A1 |
| LXR/RXR Activation | 0.00005 | AGT,ALB,APOA1,APOC3,APOE,APOH,APOM,C4A/C4B,ECHS1,HADH,IL1RL1,ITIH4,MLXIPL,SERPINF2,VTN |
| Thyroid Hormone Metabolism II  (via Conjugation and/or Degradation) | 0.00007 | DIO2,UGT1A1,UGT1A4,UGT1A6,UGT1A7,UGT2A3,UGT2B7,UGT3A1 |
| Putrescine Degradation III | 0.0001 | ALDH1B1,ALDH2,ALDH3B1,ALDH4A1,ALDH7A1,SAT2 |
| Androgen Biosynthesis | 0.00017 | AKR1C3,CYP17A1,GSTA1,HSD17B14,HSD3B2 |
| Tryptophan Degradation III  (Eukaryotic) | 0.00017 | ACMSD,EHHADH,HAAO,HADH,KMO,KYNU |
| Ethanol Degradation IV | 0.00017 | ACSS2,ALDH1B1,ALDH2,ALDH3B1,ALDH4A1,ALDH7A1 |
| Histamine Degradation | 0.00032 | ALDH1B1,ALDH2,ALDH3B1,ALDH4A1,ALDH7A1 |
| Tyrosine Degradation I | 0.00051 | FAH,HGD,HPD |
| Fatty Acid α-oxidation | 0.00074 | ALDH1B1,ALDH2,ALDH3B1,ALDH4A1,ALDH7A1 |
| Glucocorticoid Biosynthesis | 0.00079 | CYP17A1,CYP21A2,GSTA1,HSD3B2 |
| Antioxidant Action of Vitamin C | 0.00087 | GLRX,HMOX1,NOTUM,PLA2G12B,PLA2R1,PLAAT2,PLD1,PNPLA3,SLC23A1,SLC23A3,SLC2A5,TXN |
| Retinol Biosynthesis | 0.00093 | AKR1C3,BCO1,CES1,CES2,CES3,PNPLA3,RBP5 |
| Maturity Onset Diabetes of Young (MODY) Signaling | 0.00093 | ALDOB,CACNA1E,FABP1,HNF1A,HNF4A |
| Tryptophan Degradation to 2-amino-3-carboxymuconate Semialdehyde | 0.001 | HAAO,KMO,KYNU |
| NAD biosynthesis II (from tryptophan) | 0.00112 | HAAO,KMO,KYNU,QPRT |
| Acyl-CoA Hydrolysis | 0.00112 | ACOT1,ACOT4,ACOT7,HNF4A |
| Fatty Acid β-oxidation I | 0.00112 | ACAA1,ACSBG2,ECHS1,EHHADH,HADH,SLC27A2 |
| β-alanine Degradation I | 0.00145 | ABAT,ALDH6A1 |
| Choline Degradation I | 0.00145 | ALDH7A1,CHDH |
| Neuroprotective Role of THOP1  in Alzheimer's Disease | 0.00151 | ACE,AGT,CFD,DPP4,KLK1,KLK6,KLK7,MAPT,MASP1,MME,PLG,TAC1 |
| Stearate Biosynthesis I (Animals) | 0.00162 | ACOT1,ACOT4,ACOT7,ACSBG2,CYP4A22,HNF4A,SLC27A2 |
| Complement System | 0.00245 | C4A/C4B,CFD,CFI,CR1,CR2,MASP1 |
| VDR/RXR Activation | 0.00269 | CALB1,CYP27B1,IGFBP1,IGFBP6,IL1RL1,KLK6,SPP1,TRPV5,WT1 |
| Eicosanoid Signaling | 0.00339 | AKR1C3,DPEP1,GGT1,PLA2G12B,PLA2R1,PLAAT2,PNPLA3,PTGIS |
| Acute Phase Response Signaling | 0.00347 | AGT,ALB,APOA1,APOH,C4A/C4B,CRABP1,FTL,HAMP,HMOX1,HNF1A,IL6R,ITIH4,PLG,RBP5,SERPINF2 |
| Sucrose Degradation V (Mammalian) | 0.0038 | ALDOB,GALM,KHK |
| Methylglyoxal Degradation III | 0.00407 | AKR1A1,AKR1C1/AKR1C2,AKR1C3,CYP4A22 |
| Uracil Degradation II (Reductive) | 0.00417 | DPYS,UPB1 |
| Proline Degradation | 0.00417 | ALDH4A1,PRODH2 |
| D-glucuronate Degradation I | 0.00417 | AKR1A1,DCXR |
| Methionine Salvage II (Mammalian) | 0.00417 | BHMT,BHMT2 |
| Thymine Degradation | 0.00417 | DPYS,UPB1 |
| Thiosulfate Disproportionation III  (Rhodanese) | 0.00417 | MPST,TST |
| Dopamine Degradation | 0.00501 | ALDH1B1,ALDH2,ALDH3B1,ALDH4A1,ALDH7A1 |
| Mineralocorticoid Biosynthesis | 0.00708 | CYP21A2,GSTA1,HSD3B2 |
| Phenylalanine Degradation I (Aerobic) | 0.00813 | PAH,QDPR |
| Aryl Hydrocarbon Receptor Signaling | 0.00832 | ALDH1B1,ALDH2,ALDH3B1,ALDH4A1,ALDH6A1,ALDH7A1,ALDH8A1,CYP1A1,GSTA1,HSPB7,MGST1,NQO2 |
| Triacylglycerol Degradation | 0.00832 | ABHD6,CES1,CES2,CES3,NOTUM,PNPLA3 |
| Guanosine Nucleotides Degradation III | 0.00912 | GDA,NT5C1A,NT5E |
| Phospholipases | 0.00955 | HMOX1,NOTUM,PLA2G12B,PLA2R1,PLAAT2,PLD1,PNPLA3 |
| TCA Cycle II (Eukaryotic) | 0.01202 | ACO1,DHTKD1,OGDHL,SUCLG1 |
| Lysine Degradation V | 0.01318 | ALDH7A1,PIPOX |
| Citrulline-Nitric Oxide Cycle | 0.01318 | ASL,ASS1 |
| dTMP De Novo Biosynthesis | 0.01318 | SHMT1,TYMS |
| Phenylalanine Degradation IV  (Mammalian, via Side Chain) | 0.01445 | ALDH2,HPD,SLC27A2 |
| Isoleucine Degradation I | 0.01738 | ACADSB,ECHS1,EHHADH |
| Superpathway of Citrulline Metabolism | 0.01738 | ASL,ASS1,PRODH2 |
| Arginine Biosynthesis IV | 0.0195 | ASL,ASS1 |
| Urea Cycle | 0.0195 | ASL,ASS1 |
| Glycerol Degradation I | 0.0195 | GK,GPD1 |
| Glutamate Receptor Signaling | 0.02042 | GRIA3,GRM3,SLC17A2,SLC17A8,SLC1A1,SLC1A7 |
| Vitamin-C Transport | 0.02089 | GLRX,SLC23A1,TXN |
| Superpathway of Serine and Glycine Biosynthesis I | 0.0263 | PSAT1,SHMT1 |
| Purine Nucleotides Degradation II  (Aerobic) | 0.02884 | GDA,NT5C1A,NT5E |
| Glutathione-mediated Detoxification | 0.03162 | ANPEP,GGH,GSTA1,MGST1 |
| Retinoate Biosynthesis I | 0.03467 | AKR1C3,ALDH8A1,DHRS4,RBP5 |
| Methylthiopropionate Biosynthesis | 0.03802 | ADI1 |
| Glutamate Removal from Folates | 0.03802 | GGH |
| 2-amino-3-carboxymuconate Semialdehyde Degradation to Glutaryl-CoA | 0.03802 | ACMSD |
| Sorbitol Degradation I | 0.03802 | SORD |
| Folate Transformations I | 0.04365 | MTHFS,SHMT1 |
| Pyrimidine Deoxyribonucleotides  De Novo Biosynthesis I | 0.04898 | AK4,AK5,TYMS |
